# Factors Influencing the Safety and Efficiency of Antifungal Prophylaxis with Posaconazole in Children with Hematological Diseases: From Genetics to Polypharmacotherapy

**DOI:** 10.1007/s12288-019-01134-5

**Published:** 2019-05-14

**Authors:** Beata Sienkiewicz-Oleszkiewicz, Kamila Urbańczyk, Mateusz Stachowiak, Anna Rodziewicz, Aleksander Zięba, Krzysztof Kałwak, Anna Wiela-Hojeńska

**Affiliations:** 1 Department of Clinical Pharmacology, Faculty of Pharmacy, Wrocław Medical University, 211a Borowska St., 50-556 Wrocław, Poland; 2 Department of Paediatric Bone Marrow Transplantation, Oncology and Hematology, Wrocław Medical University, 213 Borowska St., 50-556 Wrocław, Poland

**Keywords:** ABCB1 protein, Adverse drug event, Children, Posaconazole, Hematology

## Abstract

The aim of this study was to determine the impact of ABCB1 polymorphism, BMI, age and drug co-administration on safety and efficiency of posaconazole (PCZ) oral suspension treatment in children with hematological diseases. Seventy children were included in the study. ABCB1 polymorphism in fifty-eight children was determined using a PCR–RFLP method. A protocol with data on the health condition, treatment and adverse events (AE), as well as a survey on treatment tolerance for the legal guardians was evaluated. Liver function tests were observed for the first 20 days, and AE during the complete medication period. For statistical analysis a χ^2^ test with Yates’s correction, a Pearson’s or Spearman’s correlation test was performed (*p* < 0.05). Genetic testing showed 24% CC, 46% CT and 30% of TT variants. PCZ prophylaxis failed in twenty cases, where change in prophylactic treatment was needed. Fifty-two children suffered from at least one mild to moderate adverse event. Sixty-five legal guardians completed the survey, most of them reported the treatment to be well tolerated. ABCB1 polymorphism had no impact on AE occurrence and posaconazole prophylaxis efficiency. Age influenced the number of gastrointestinal (*p* = 0.02), visual (*p* = 0.05), neurological (*p* = 0.01), dermatological (*p* = 0.002) and flu-like (*p* = 0.02) complications. AST (*p* = 0.03) and LDH (*p* = 0.008) activity presented age dependency. The concomitant use of proton pump inhibitors (PPI) had impact on liver health parameters elevation (*p* = 0.009) and circulatory system complications (*p* = 0.008). High incidence of mild to moderate AE, and other factors influencing PCZ pharmacokinetics (PPI co-administration, obesity), suggest a need for careful pediatric onco-hematology patient evaluation.

## Introduction

Posaconazole (PCZ) is a triazole antifungal agent. The drug is primarily indicated for molds infection prophylaxis in patients receiving chemotherapy for acute myeloid leukemia (AML), myelodysplastic syndrome (MDS) and in patients after allogenic hematopoietic stem cell transplantation (allo-HSCT).

It may also be used for treatment of particular cases of invasive aspergillosis, fusariosis, chromoblastomycosis, coccidioidomycosis and candidiasis [1, 2].

The treatment of children is very specific. Not only from the psychological point of view, also the pharmacokinetic properties of drugs differ from the adult population. Basic alterations are gathered in Table 1 [3, 4].

**Table 1:** Factors influencing drug pharmacokinetics in pediatric population [3, 4]

Absorption	Distribution	Metabolism	Excretion
↓ Surface of gastrointestinal tract ↓ HCl secretion ↓ Motility and peristalsis ↓ Gastric emptying ↓ Bile secretion Immature enzymes Thinner stratum corneum ↑ Hydration of epidermidis Variable skeletal muscle blood flow	↑ Total body water ↑ Extracellular water ↓ Fat content ↑ Body water: fat ratio ↓ Protein serum levels, especially albumins ↓ α_1_-Glycoprotein concentration ↓ Protein binding ↑ Permeability of brain-blood barrier	↓ CYP3A7 after birth, barely measurable in adults ↓ CYP2D6 (20% of adult activity at 1 month of postnatal age, adult competence—3–5 years of age) ↓ CYP2C9, CYP2C19 (low activity during the first week of life, adult activity—6 months of age) ↓ CYP1A2 (adult levels—4 months of age, in children 1–2 years of age may be exceeded) ↓ CYP3A4 (low activity in the first month of life, adult levels—6–12 months postnatally) ↓ *N*-acetyltransferase 2 (NAT2) (adult activity 1–3 years of age) ↓ Uridine diphoshoglucuronyltransferase (UDP-GT) (adult activity—6–18 months of age)	↓ Glomerular filtration rate ↓ Renal tubular absorption

According to the Food and Drug Administration (FDA) PCZ oral formulation is approved for children older than 13 [2, 3]. Some studies showed that the agent is also safe in children younger than 12 [5]. Although posaconazole has either no grading (in cases of children after allo-HSCT without GVHD) or a BI grade (in children after allo-HSCT with GVHD and in de novo or recurrent leukemias) according to the *Recommendations for primary chemoprophylaxis of invasive fungal diseases in paediatric patients with cancer or haemopoietic stem*-*cell transplantation* of the ECIL-4, it is often used in clinic for antifungal prophylaxis in children with hematological malignances [6].

The pediatric onco-hematology population is particularly exposed to adverse drug reactions (ADR) and adverse events (AE). The effectiveness and safety of PCZ oral suspension is influenced mainly by food intake, gastric pH and motility as well as drug-drug interactions [5, 7, 8]. Posaconazole is a substrate for UDP-glucuronosyltransferase and P-glycoprotein, thus agents having impact on these two clearance pathways must be taken into consideration during PCZ medication [1].

As P-glycoprotein is encoded by the ABCB1 gene, the polymorphism of which is connected with changes in the protein expression, the aim of our study was to determine the impact of ABCB1 polymorphism on the safety and efficiency of posaconazole oral suspension treatment in children. We also took into consideration other agents potentially influencing antifungal prophylaxis (BMI, age, drug co-administration). To our knowledge it is the first of this kind study performed among the Polish pediatric population with hematological diseases.

## Materials and Methods

The study was performed according to the Declaration of Helsinki and with approval of the Bioethics Committee of the Wrocław Medical University (KB-657/2012). It is a single center, non-randomized retrospective analysis of seventy pediatric patients who received posaconazole oral suspension for antifungal prophylaxis.

### Patients Characteristics

The included children were patients of the Paediatric Bone Marrow Transplantation, Oncology and Hematology Unit, of the Wrocław Medical University Hospital, aged between 2 months and 19 years. The median PCZ dose was 8 mg per kg of body weight, two times a day according to the algorithm evaluated by Welzen et al. [9]. Most important data on patients enrolled is presented in Table 2.

**Table 2:** Patient characteristics

Characteristic	Number of patients (%)
Gender	
Male	43 (61.4)
Female	27 (38.6)
Age (median) [years] − median (25Q–75Q)	7 (2.0–14.0)
< 6	32 (45.7)
7–11	14 (20)
> 12	24 (34.3)
Diagnosis	
ALL	23 (32.8)
AML	18 (25.7)
SAA	8 (11.4)
WAS	4 (5.7)
Other hematological diseases	16 (24.4)
BMI [kg/m^2^] − median (25Q–75Q)	16.4 (14.3–18.6)
< 18.5	51 (72.8)
18.5–25	17 (24.3)
> 25	2 (2.9)

*ALL* acute lymphoid leukemia, *AML* acute myeloid leukemia, *SAA* serious aplastic anemia, *WAS* Wiskott-Aldrich Syndrome

### Patients’ Protocol and Survey

A protocol with most important data on patients health condition, underlying diseases, allo-HSCT performance, previous infections, treatment and adverse events was evaluated. As posaconazole may lead to hepatic impairment we also monitored biochemical parameters of liver function. Those were aspartate aminotransferase (AST), alanine transaminase (ALT), activated partial thromboplastin time (APTT), gamma-glutamyltransferase (GGT), lactate dehydrogenase (LDH) activity, albumin and fibrinogen concentrations at three time points (before, at the seventh, and the twentieth day) of PCZ prophylaxis.

Drug-drug interactions are also factors influencing PCZ pharmacokinetics and inducing AE occurrence. So, we tried to determine, on the basis of patients treatment history, drugs potentially interacting with the antifungal agent. We monitored AE during the complete posaconazole conducted medication period.

In addition a survey on treatment tolerance and observed adverse events was evaluated and performed among the legal guardians of the examined children. Sixty-five of them completed the questionnaire.

The survey consisted of five parts: first—connected with basic information (body weight (BW), age, height), second—with information on previous fungal infections (occurred before hospital admission), third—potential food supplement administration, fourth—adverse events observed during posaconazole treatment (we intentionally listed AE possibly occurring during PCZ therapy) and fifth—concerning the treatment satisfaction in a 0–5 score scale. On this scale 0 meant no opinion, 1-no satisfaction (serious fungal infection occurred despite posaconazole prophylaxis), 2-low satisfaction (local fungal infection in more than one body area occurred), 3-moderate satisfaction (local fungal infection occurred), 4-satisfaction (no fungal infection, but adverse events occurred), 5-full satisfaction (no fungal infection and good tolerance).

### DNA Isolation and Genetic Testing

We collected seventy samples (one from each patient) of whole blood drawn on EDTA. The samples were stored at -20 °C.

DNA was isolated using the QIAamp^®^ DNA Blood Mini Kit according to the manufacturer instruction, in a laminar flow cabinet.

For ABCB1 polymorphism determination, an adapted Siegmund et al. [10] polymerase chain reaction-restriction length polymorphism (PCR–RFLP) method was used.

### Statistical Analysis

We performed statistical analysis on the collected data. For every group the number of cases (N), median (M), range (min–max), upper and lower quartile (25Q-75Q) was calculated. The hypothesis on the equality of median values of the groups was verified using the Kruskal–Wallis test.

For discrete parameters the frequency of features in the groups was analyzed using the Chi squared test with Yates’s correction, with an adequate number of degrees of freedom *df* (*df* = (m − 1) * (n − 1)).

For chosen pairs of parameters a Pearson’s or Spearman’s correlation test was performed.

*p* values less than 0.05 were considered statistically significant. *p* values between 0.05 and 0.1 were considered as tendency. The statistical analysis was performed using the EPIINFO program (Ver. 7.1.1.14, 2013, USA).

## Results

Seventy patients of the Paediatric Bone Marrow Transplantation, Oncology and Hematology Unit, of the Wrocław Medical University Hospital were included in our study. The median BMI was 16.4 kg/m^2^, two children were overweight. During the observation period 21 (30%) children suffered from acute GVHD, seven of them further developed a chronic form of the disease.

Fifty-eight legal guardians agreed for genetic testing of their children. The performed C3435T genetic testing for ABCB1 polymorphisms showed that 24% (N = 14) of patients presented the CC, 46% (N = 27) the CT, and 30% (N = 17) the TT variant.

Posaconazole oral formulation prophylaxis failed in 20 cases, where a change in treatment was needed according to suspected IFI development.

**Fig. 1:**
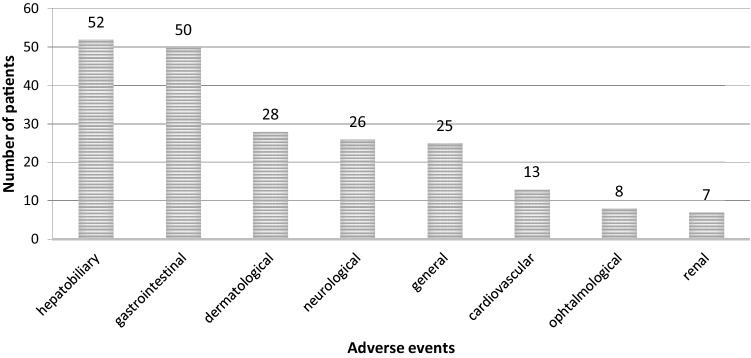
Adverse events considered with PCZ prophylaxis in children

Fifty-two children suffered from at least one mild to moderate adverse event during invasive fungal infection prophylactic treatment. However according to the performed survey legal guardians reported the treatment to be well tolerated by their children (number of completed surveys 65; score 5 N = 21, score 4 N = 21, score 3 N = 10, score 2 N = 2, score 1 N = 0, score 0 N = 11). There was no need to discontinue posaconazole prophylaxis due to adverse events in any case. The occurrence of AE among different systems is presented in Fig. 1.

Increased AST activity was observed in 35 cases, ALT activity increased in 42, GGT in 28 cases. Nine children presented higher bilirubin values. Most frequent gastrointestinal disturbances were vomiting (N = 29), nausea (N = 25), loss of appetite (N = 22), diarrhea (N = 21), constipation (N = 20) and abdominal pain (N = 20). Dermatological complication manifested as mouth ulceration (N = 24) and rash (N = 11). Nervous system disorders showed as headache (N = 10), insomnia (N = 7) and tremor (N = 5). Ophthalmological disorders most often were connected with blurred vision (N = 9) whereas cardiovascular disorders with hypertension (N = 6). Renal disorders manifested as blood urea concentration increase in 5 and hemorrhagic cystitis in 2 cases.

Drug-drug interaction analysis led to determination of agents potentially inducing AE occurrence during PCZ treatment, having impact on its pharmacokinetic properties. Drugs and number of children treated are presented in Table 3.

**Table 3:** Drugs potentially interacting with posaconazole during concomitant use

Drug/pharmacologic group	Number of children receiving the drug (%)
Calcineurin inhibitors	52 (74.3)
Biseptol	52 (74.3)
Acyclovir	50 (71.4)
Analgetics	40 (57.1)
Methotrexate	39 (55.7)
Ciprophloxacin	33 (47.1)
Proton pump inhibitors	20 (28.6)
Antiemetic’s	20 (28.6)
Vincristine	9 (12.9)
Amlodypine	5 (7.1)
Anxiolytics	3 (4.3)
Fluconazole	2 (2.9)

Statistical analysis showed that ABCB1 polymorphism has no impact on overall adverse event occurrence and posaconazole prophylaxis efficiency as presented in Table 4. However two patients with good tolerance to posaconazole, who did not suffer from any adverse event during antifungal treatment presented the CC variant. We found a tendency for APTT prolongation during treatment in individuals presenting the TT variant (*p* = 0.07, χ^2^ = 8.45). After twenty days of posaconazole treatment the APTT prolonged in four patients presenting the CT variant (N = 27, 14.8%), one patient with CC variant (N = 14, 0.07%) and in 7 patients presenting the TT variant (N = 17, 41.2%).

**Table 4:** Influence of ABCB1 polymorphism on adverse events occurrence during posaconazole prophylaxis

Adverse event	CT N = 27 (46%)		TT N = 17 (30%)		CC N = 14 (24%)		P (χ^2^)
	0	1	0	1	0	1	
Gastrointestinal disturbances	7	20	5	12	4	10	0.964 (0.0724)
Ophthalmological disturbances	22	5	16	1	13	1	0.369 (1.99)
Elevation of liver function parameters	3	24	3	13	4	9	0.313 (2.32)
Neurological disturbances	16	11	11	6	10	4	0.741 (0.600)
Dermatological changes	16	11	9	8	9	5	0.812 (0.416)
Renal function changes	25	2	15	2	12	2	0.770 (0.522)
General symptoms	17	10	10	7	10	4	0.762 (0.543)
Cardiovascular disturbances	21	6	13	4	12	2	0.790 (0.472)
Invasive fungal infection	21	6	13	4	7	7	0.148 (3.821)

0—no adverse event occurred

1—adverse event occurred

The age of children influenced the number of gastrointestinal, visual, neurological, dermatological and flu-like complications. The older the child the more often mentioned AE occurred. Furthermore AST and LDH activity presented age dependency especially in the first week of PCZ treatment.

Children with higher BMI values were more susceptible for visual and skin disturbances (*p* = 0.057, H = 3.61) as well as increased LDH activity during the first week of IFI prophylaxis.

Gender did not influence AE frequency.

The concomitant use of proton pump inhibitors (PPI) had great impact on parameters for liver health elevation and circulatory system complications during PCZ prophylaxis.

Most important findings on variables influencing adverse events during posaconazole conducted prophylaxis and their statistical significance are presented in Table 5.

**Table 5:** Most important findings on variables influencing adverse events during posaconazole treatment and their statistical significance

Variable potentially influencing adverse event occurrence during PCZ treatment	Age
Impact on safety of PCZ treatment	Yes
Number of patients tested (%)	70 (100%)
Test	Spearmans r = 0.45 *p* value = 0.00009

	Number of patients (%)	Spearmans r	*p* value
Additional information on adverse events			
Higher AST activity during first week of treatment	18 (26%)	− 0.27	0.036
Higher LDH values during first week of treatment	13 (19%)	− 0.35	0.008

	Number of patients without/with AE	No adverse events occured median (25Q–75Q)	Adverse events occured median (25Q–75Q)	Kruskal–Wallis H	*p* value
The older the child the more often following adverse events occur					
Gastrointestinal	20/50	3.15 (1.42–8.50)	9.00 (3.70–15.0)	5.06	0.0244
Neurological	44/26	5.90 (1.73–11.0)	13.0 (6.0–15.4)	5.71	0.0168
Dermatological	42/28	4.25 (1.10–11.0)	12.5 (6.5–15.4)	9.32	0.0023
Flu-like	45/25	4.50 (1.70–13.0)	10.0 (6.0–15.2)	4.74	0.0295
Visual	62/8	6.50 (1.90–14.0)	13.5 (9.50–15.4)	3.72	0.0536

Variable potentially influencing adverse event occurrence during PCZ treatment	BMI
Impact on safety of PCZ treatment	Yes
Number of patients tested (%)	70 (100%)
Test	Spearmans r = 0.24 *p* value = 0.47

	Number of patients (%)	Spearmans r	*p* value
Additional information on adverse events			
Increased LDH activity during first week of treatment	13 (19%)	− 0.35	0.01

Variable potentially influencing adverse event occurrence during PCZ treatment	Concomitant PPI administration
Impact on safety of PCZ treatment	Yes
Number of patients tested (%)	64 (91%)

	Number of patients (%)	χ^2^	*p* value
Additional information on adverse events			
Elevations in liver function test	20 (31%)	6.71	0.009
Circulatory system complications	8 (13%)	6.97	0.008

Variable potentially influencing adverse event occurrence during PCZ treatment	ABCB1 polymorphism
Impact on safety of PCZ treatment	No
Number of patients tested (%)	58 (83%)

	Number of patients (%)	χ^2^	*p* value
Additional information on adverse events			
Tendency for APTT prolongation in patients presenting the TT variant	7 (41%)	8.45	0.076

Variable potentially influencing adverse event occurrence during PCZ treatment	Concomitant cyclosporine administration
Impact on safety of PCZ treatment	No
Number of patients tested (%)	68 (97%)
Test	χ^2^ = 8.75 *p* value = 0.271

Variable potentially influencing adverse event occurrence during PCZ treatment	Gender
Impact on safety of PCZ treatment	No
Number of patients tested (%)	70 (100%)
Test	χ^2^ = 2.61 *p* value = 0.919

## Discussion

The aim of this investigation was to determine the impact of ABCB1 polymorphism, BMI, age and drug co-administration on safety and efficiency of posaconazole oral suspension treatment in children with hematological diseases.

In our study the frequencies of ABCB1 C3435T genetic variants (24% for CC, 46% for CT and 30% for the TT variant) were comparable with other research performed among the Polish hematology population [11, 12].

We evaluated the influence of ABCB1 polymorphism on adverse event occurrence in children with hematological malignances treated with PCZ oral suspension. We found no statistical correlation, however a tendency for APTT prolongation during treatment was observed. This finding is completely new and to our knowledge has not been observed in any research before.

What is also interesting in our study, is that patients with good tolerance to posaconazole and with proper prophylactic response (N = 2) presented the CC variant. This may be connected with higher activity of P-gp in patients with this ABCB1 genetic polymorphism, that was previously reported in patients with B cell chronic lymphocytic leukemia, and what comes along with the increased active transport of drugs out of cells, suggesting and supporting the thesis of a protective role of the C3435C variant [12].

We didn’t find a correlation between ABCB1 polymorphism and efficiency of PCZ prophylaxis measured as success or failure of prophylactic treatment. To our knowledge we were the first to evaluate this problem.

During posaconazole pharmacotherapy relatively high incidence of mild to moderate adverse events occurred. Most frequent were gastrointestinal, visual, neurological, dermatological and flu-like complications. The observations are concise with those from other studies. Lehrnbecher et al. [13] reported fever, nausea and/or vomiting, abdominal pain, diarrhea, headache, and skin eruptions as most common during PCZ treatment. Gastrointestinal and skin adverse events, according the observations of Döring et al. [5], were the most common complications.

The elevation of liver function tests namely transaminase activity and LDH activity is a well-known adverse drug reaction caused by triazole antifungal agents [1, 5, 13–15]. What is a new finding, and as to our knowledge was not observed in any of the previous studies in children, is a statistically significant correlation between age and mentioned AE. The older the child the more often described adverse events occurred. A similar tendency was observed for adults, where a reduction in the apparent volume of distribution, and 11% higher average plasma posaconazole concentration were observed according to older age [16]. However as children generally have different pharmacokinetics than adults, this finding cannot be directly extrapolated into the pediatric population.

The concomitant use of proton pump inhibitors (omeprazole, pantoprazole) had impact on parameters for liver health and circulatory system complications during PCZ prophylaxis. The influence of concomitant PPI administration with posaconazole is well established for the adult population and was also evaluated in children [7, 16–19]. Although in general the PPI co-administration is connected with lower posaconazole serum concentrations, during our study we observed that adverse events occurred more often in patients receiving omeprazole or pantoprazole. This could be connected with other drugs used for hematological malignancy treatment. PPI—especially the older representatives (omeprazole, pantoprazole) are CYP2C19 substrates, and therefore might affect pharmacokinetics of other drugs metabolized through CYP2C19 [20].

We observed that children with higher BMI values are more susceptible for visual and skin disturbances as well as increased LDH activity during the first week of IFI prophylaxis conducted with posaconzole. This problem was not evaluated for the pediatric population receiving PCZ in oral suspension formulation. However there is one study describing the experience from using posaconazole in form of extended released tablets. In obese adult patients (BMI > 30 kg/m^2^, and body weight > 90 kg) the antifungal agents serum levels were lower during the tablet formulation treatment [21]. Our observations are not concise with this finding. This could be due to alterations of pharmacokinetic properties of both formulations and both populations (e.g. in children the glucuronidation potential is not as high as in adults, thus it cannot be increased in childhood obesity, as it is the case in the adult population).

## Conclusion

ABCB1 polymorphism has no impact on the efficiency and safety of posaconazole treatment in children with hematological malignancies. However the relatively high incidence of mild to moderate adverse events during prophylaxis, and occurrence of other factors potentially influencing PCZ pharmacokinetics such as PPI co-administration or obesity, suggest a need for careful patient evaluation and probable TDM (therapeutic drug monitoring) enrollment in onco-hematological pediatric patients receiving antifungal prophylactic treatment conducted with posaconazole oral suspension formulation.

What is certainly a limitation of our study is the relatively small group of patients and lack of posaconazole serum concentration measurement. However the aim of our retrospective research was to evaluate potential causes of AE occurrence, and prophylaxis failure during PCZ treatment. As our findings were partially new there is certainly a need for larger studies to evaluate the problem in the future.
